# HAR_Locator: a novel protein subcellular location prediction model of immunohistochemistry images based on hybrid attention modules and residual units

**DOI:** 10.3389/fmolb.2023.1171429

**Published:** 2023-08-17

**Authors:** Kai Zou, Simeng Wang, Ziqian Wang, Zhihai Zhang, Fan Yang

**Affiliations:** ^1^ School of Communications and Electronics, Jiangxi Science and Technology Normal University, Nanchang, China; ^2^ Artificial Intelligence and Bioinformation Cognition Laboratory, Jiangxi Science and Technology Normal University, Nanchang, China

**Keywords:** hybrid attention modules, residual units, multi-view abstract features, protein subcellular location prediction, immunohistochemistry images

## Abstract

**Introduction:** Proteins located in subcellular compartments have played an indispensable role in the physiological function of eukaryotic organisms. The pattern of protein subcellular localization is conducive to understanding the mechanism and function of proteins, contributing to investigating pathological changes of cells, and providing technical support for targeted drug research on human diseases. Automated systems based on featurization or representation learning and classifier design have attracted interest in predicting the subcellular location of proteins due to a considerable rise in proteins. However, large-scale, fine-grained protein microscopic images are prone to trapping and losing feature information in the general deep learning models, and the shallow features derived from statistical methods have weak supervision abilities.

**Methods:** In this work, a novel model called HAR_Locator was developed to predict the subcellular location of proteins by concatenating multi-view abstract features and shallow features, whose advanced advantages are summarized in the following three protocols. Firstly, to get discriminative abstract feature information on protein subcellular location, an abstract feature extractor called HARnet based on Hybrid Attention modules and Residual units was proposed to relieve gradient dispersion and focus on protein-target regions. Secondly, it not only improves the supervision ability of image information but also enhances the generalization ability of the HAR_Locator through concatenating abstract features and shallow features. Finally, a multi-category multi-classifier decision system based on an Artificial Neural Network (ANN) was introduced to obtain the final output results of samples by fitting the most representative result from five subset predictors.

**Results:** To evaluate the model, a collection of 6,778 immunohistochemistry (IHC) images from the Human Protein Atlas (HPA) database was used to present experimental results, and the accuracy, precision, and recall evaluation indicators were significantly increased to 84.73%, 84.77%, and 84.70%, respectively, compared with baseline predictors.

## 1 Introduction

Proteins are important biomacromolecules at the eukaryotic cellular level and are delivered to appropriate subcellular compartments where they interact with other biomolecules. Especially, several diseases have been reported to be significantly associated with the subcellular location of protein expression. For instance, Nucelolin in several subcellular locations, such as nucleolus, nucleoplasm, cytoplasm, and cell membrane impacts cancer development and therapy (Berger et al., 2015) and the loss of BRCA1 in nuclear or cytoplasmic is observed as a marker of breast tumor aggressiveness (Madjd et al., 2011). Therefore, understanding the subcellular locations of proteins is conducive to analyzing the functional principle of proteins, comprehending the complex physiological reaction process, and finding out the cancer biomarker (Kumar et al., 2014; Thul et al., 2017; Cheng et al., 2019; Shen et al., 2021). The classical solution that wet-experiment observation has been used to identify protein subcellular location, which exposes low-efficiency, time-consuming and labor-intensive with an increasing number of proteins. A resolution to these issues is highly desired, with one intriguing option being automated high-performance predictors, which have been explored to speed up the study of protein subcellular location (Yu et al., 2006; Chung, 2010; Du, 2017).

At present, protein expression patterns that are used to study protein subcellular location prediction systems fall into two types: amino acid molecular sequence and microscopic image. The former represents the similarity of subcellular locations between proteins by quantifying the intermolecular correlation of one-dimensional amino acid sequences (Shi et al., 2007; Nair and Rost, 2009; Shen and Chou, 2009; Cheng et al., 2018; Sun and Du, 2021). The latter expresses the dependability of subcellular locations between unknown and known proteins by applying advanced image processing technology to extract two-dimensional image properties (Chen and Murphy, 2006; Newberg and Murphy, 2008; Coelho et al., 2013; Xu et al., 2013). By comparison, sequence-based systems have higher accuracy but cannot reflect changes in the biochemical environment of tissues; On the other hand, some methods founded on microscopic images provided researchers with several image views of protein regions, such as shape, outline, texture, and cell distribution information, which helps to capture changes in the physiological environment to screen pathological tissues and cancer biomarkers (Uhlen et al., 2017). Particularly, with the development of machine learning and deep learning, considerable progress has been inspired by using hand-crafted features obtained from featurization or abstract features derived from representation learning in image-based models (Bengio et al., 2013; Shao et al., 2017; Xu et al., 2018).

With respect to featurization or hand-crafted features, shallow features are introduced to describe an image's global or local numerical information by statistical methods. For instance, Subcellular Location Features (SLFs) were employed to describe the shallow features of microscope images at the global level, including morphological features, Zernike moment features, Haralick texture features, and wavelet features (Newberg and Murphy, 2008). Zernike moment features, obtaining descriptive features by applying orthogonal Zernike polynomials to a unit circle of the set of complex functions, have been adopted to express rotational invariance properties on images (Boland and Murphy, 2001; Huang and Murphy, 2004; Chebira et al., 2007; Chen et al., 2007). Haralick features were adopted to quantitatively describe inertia and isotropy of intuitive pattern of protein subcellular location relying on omni-directional Gray-Level Co-occurrence Matrix (GLCM) (Xu et al., 2013). In addition, DNA distribution information, which means protein and nuclear object overlap and distance, was deployed to supplement global information since each protein image was accompanied by nuclear information (Bengio et al., 2013; Liu et al., 2019; Xue et al., 2020; Su et al., 2021; Ullah et al., 2021). An image intensity coding strategy was utilized to quantize frequency features in the frequency domain space of image wavelet transforms, which was conducive to releasing sparsity problems of immunohistochemistry (IHC) images and strengthening discriminative ability (Yang et al., 2019). To further decrypt images from multi-view, local-level information was powerfully presented to supplement global information. Local Binary Patterns (LBP), Local Ternary Patterns (LTP), and Local Quinary Patterns (LQP) are grounded on the statistic of the histogram between the center and surrounding pixels to express local texture information (Xu et al., 2013; Yang et al., 2014). Speeded-Up Robust Features (SURF) were derived from local operator features by detecting interesting points using an approximate Gaussian blob detector in both space and scale (Coelho et al., 2013). The structural relationship among cellular components as effective prior information has been considered advanced in the protein subcellular image feature space by combining Haralick and LBP features (Shao et al., 2016). Although the above image feature properties have been effectively validated, the inherently weak supervisory properties and poor distinctness of shallow features have been limiting the further improvement of model performance.

Unlike handcrafted features such as time domain features and frequency transformation features, the representation learning that learning representation of IHC images based on deep learning makes it easier to extract more supervisory and representational information (Bengio et al., 2013). With the evolution of deep learning, predictors based on Convolutional Neural Networks (CNNs) map image feature vectors into high-dimensional space through numerous nonlinear activation functions to obtain more robust representations and produce impressive performance in many fields, such as Face Recognition, Image Recognition, and Object Detection (Szegedy et al., 2013; Simonyan and Zisserman, 2014; Sun et al., 2015). Meanwhile, various abstract features from classical deep learning models trained in a fully supervised setting have consistently proved effective on generic vision tasks, such as Person Re-identification and Human Activity Recognition (Donahue et al., 2014; Sani et al., 2017; Nie et al., 2019). Consequently, feature maps in the last or penultimate layer of pre-train CNNs were extracted and incorporated into shallow features to enhance the supervisory and distinctness of protein subcellular location in the IHC images (Shao et al., 2017; Liu et al., 2019; Xue et al., 2020; Su et al., 2021; Ullah et al., 2021). It can be explained that abstract features from deep learning models describe abstract morphological local regions, edges, corners, outlines, and other digital image characteristics and serve as a helpful supplement to texture, inertia, isotropy, and the spatial ratio of shallow features. An 11-layer neural network trained in yeast cells describes basic digital image characteristics and spate subcellular localization classes with an increasing depth of layer; besides, abstract features from the neural network have been proven useful for predicting the subcellular localization of fluorescent proteins (Pärnamaa and Parts, 2017). Abstract features obtained from a deep CNN were organized with histomorphologic information to recognize lesional coordinates of cancer tissue images (Faust et al., 2018). Abstract features from CNNs do improve the supervisory and discriminative capabilities of shallow features in digital images, but they do not account for the poor information richness and abstraction attributed to some reasons: firstly, general convolution and pooling operations are difficult to focus on the protein-target regions in bio-images; secondly, large-scale bio-images are prone to get stuck in information degradation resulting in poor performance.

In this work, a predictor named HAR_Locator based on the Hybrid Attention modules and Residual units was proposed to predict protein subcellular location in IHC images. The advancement of HAR_Locator has several attractive attributes: firstly, the features extractor known as HARnet is developed based on Hybrid Attention modules and Residual units to effectively highlight discriminating abstract features, convey the gradient information of IHC image in the network, and prevent information loss (He et al., 2016); secondly, the multi-view abstract features from different layers of HARnet were concatenated with shallow features obtained from statistical methods to improve the supervised ability of features; thirdly, the Binary Relevance (BR) and the Stepwise Discriminant Analysis (SDA) were adopted to fit feature space from concatenation; finally, a multi-category multi-classifier decision system based on ANN was utilized to output decision results from multiple confidence levels of multiple classifiers. In addition, a benchmark dataset of 6778 IHC images from HPA, including 59 proteins, was collected to verify the effectiveness of the HAR_Locator. The experimental results show that the HAR_Locator reaches 84.73%, 84.77%, and 84.70% respectively in accuracy, precision, and recall, and is significantly improved compared with other baseline predictors.

## 2 Materials and methods

### 2.1 The benchmark dataset

In this study, a benchmark dataset with a total number of 6,778 IHC images was collected from HPA (https://www.proteinatlas.org/), including 5,725 high-level stain expressions with Enhance label reliability images and 1,053 high-level stain expressions with Support label reliability images. The HPA database was created in 2005 with the goal of providing researchers with information on the expression and localization of proteins in human tissues or cells. Researchers can freely access three types of protein images, namely, immunohistochemistry (IHC) images, immunofluorescence (IF) images, and pathology (PA) images, which respectively reflect the protein information at the tissue, cell, and pathology levels. As IHC images are widely used in clinical applications and screening cancer biomarkers, this work selects IHC images as the research target. There are seven subcellular locations of proteins: Cytosol, Endoplasmic Reticulum (ER), Golgi apparatus, Nucleoli, Mitochondria, Centrosome, and Vesicles. The dataset is shown in Table 1. Hereinto, the high-level stain expression means the protein channel with the best staining in IHC images; similarly, weaker staining levels include Medium, Low, and Not detected. Enhanced label reliability refers to the annotation of proteins being validated by one or several antibodies, and proteins with the Enhance annotation are not reported in contradiction with the existing annotation in the HPA database by published literature. Support label reliability is not validated by several antibodies like Enhanced, but the annotation of subcellular localization is described in other literature. Expect for mentioned two reliability levels, there are two other lower levels of evaluation, i.e., Improve and Uncertain.

**TABLE 1:** The data volume of protein subcellular location in the dataset.

Item	Subcellular location	Number of images
Class0	Cyto.	999
Class1	ER	996
Class2	Golgi	1000
Class3	Nucl.	1000
Class4	Mito.	1000
Class5	Cent.	788
Class6	Vesi.	995

Notes: Cyto., cytosol; ER., endoplasmic reticulum; Golgi, Golgi apparatus; Nucl., nucleoli; Mito., mitochondria; Cent., centrosome; Vesi., vesicles.

### 2.2 The HAR_Locator constructed by concatenating multi-view abstract and shallow features

**FIGURE 1:**
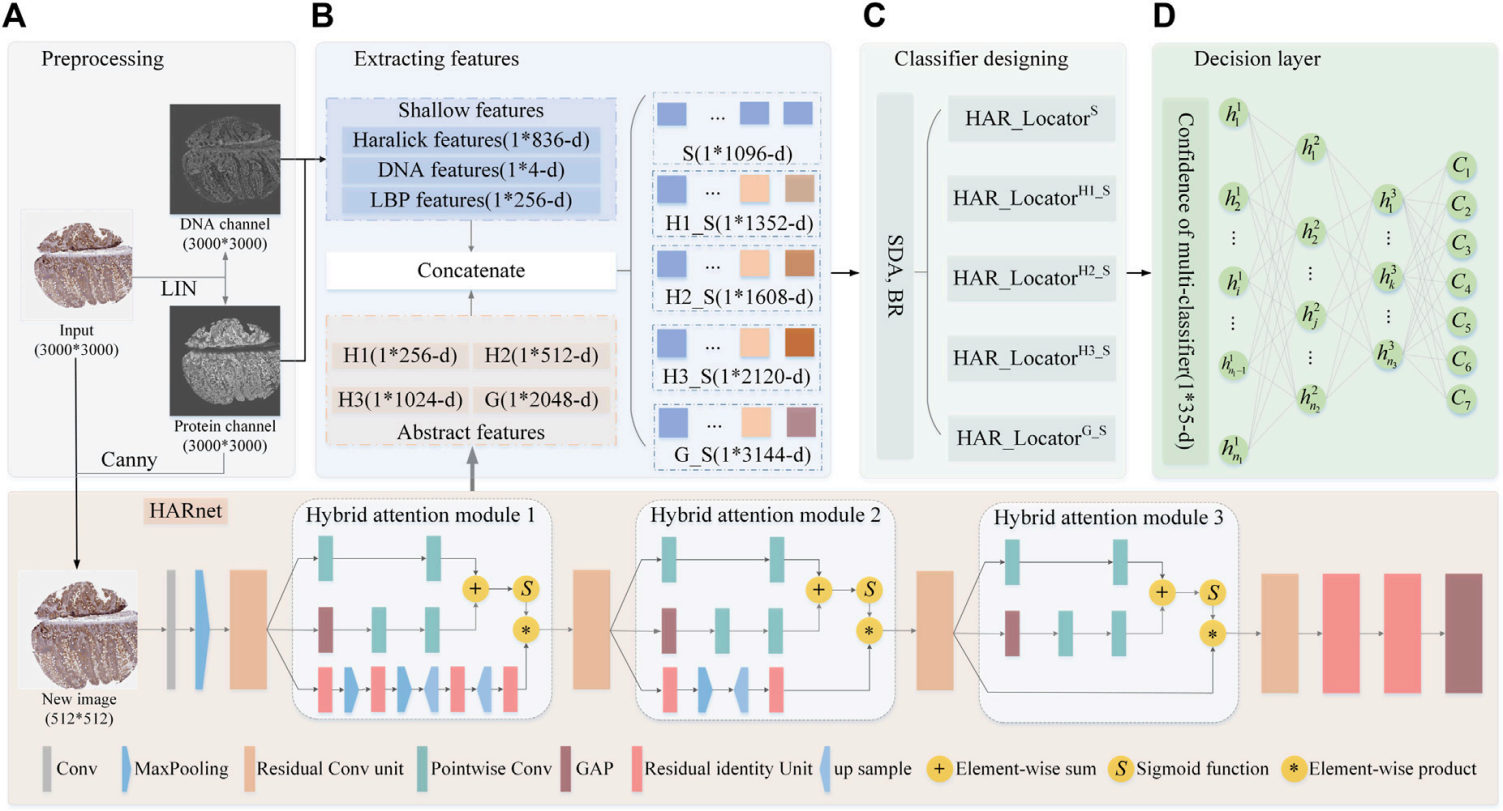
The flowchart of HAR_Locator proposed in this work. **(A)** the preprocessing of IHC images; **(B)** extracting shallow and abstract features; **(C)** fitting multiple BR classifiers in integrated features space; **(D)** outputting decision result by ANN. Abbreviation definitions: H1, Hybrid attention module 1; H2, Hybrid attention module 2; H3, Hybrid attention module 3; G, the output of the last Global Average Pooling (GAP) layer; HAR_Locator^H1_S^, the predictor was constructed by concatenating H1 features with shallow features; HAR_Locator^H2_S^, the predictor was constructed by concatenating H2 features with shallow features; HAR_Locator^H3_S^, the predictor was constructed by concatenating H3 features with shallow features; HAR_Locator^G_S^, the predictor was constructed by concatenating G features with shallow features.

The algorithm framework of HAR_Locator is shown in Figure 1, and it consists of four protocols: A, getting interesting regions by preprocessing technologies for the subsequent feature extraction; B, extracting shallow features by statistical methods and abstract multi-view features from HARnet; C, the establishment of multiple classifiers through SDA and BR classifier; D, getting decision result by the ANN. The details are covered in the next section.

#### 2.2.1 The preprocessing of IHC images

IHC images from HPA were photographed at bright field images of tissue level using an RGB camera, reflecting purple DNA in nuclei and brown protein in subcellular locations after DNA and proteins with the corresponding chemical reagent staining, whose size would be roughly 3,000 × 3,000 resolutions. In order to eliminate badly stained images, the empirical threshold filtering method was employed to delete those IHC images with bad staining quality (Newberg and Murphy, 2008). There are six images with poor staining, and 6,772 images were left after deletion. After that, IHC images were unmixed into protein and DNA channels by Linear Spectral Separation (LIN) (Newberg and Murphy, 2008), as shown in Figure 1A. LIN was employed to transform the IHC from RGB to HSV space for calculating the statistic histogram of hue value. The original IHC image was unmixed out of protein and DNA channels based on the color conversion matrix from the two peaks of the histogram. Moreover, to remove invalid border information from IHC images, the canny operator with two scale factors is used to obtain the protein region, and then it is mapped back to the original IHC image (Bao et al., 2005). After mentioned preprocessing stags, the protein and DNA channels were adopted to get shallow features by statistical methods and the new images with 512 × 512 resolutions were fed into HARnet to gain abstract features. Details are as described later.

#### 2.2.2 Global and local shallow feature operators acting on protein and DNA channels

As a classical quantitative representation method of IHC images, SLFs based on the statistical method have been proven advanced in describing global and local information (Newberg and Murphy, 2008; Xu et al., 2013). The well-known Haralick features are leveraged to describe the inertia and isotropy of intuitive patterns of protein subcellular location from a global perspective (Newberg and Murphy, 2008). Specifically, 836-dimension Haralick features of the protein channel were calculated by discrete wavelet transform using the Daubechies filter, which was extracted by calculating texture feature components in the horizon, vertical, and two diagonal directions. Furthermore, 4-dimensional DNA spatial distribution features were obtained by calculating the DNA occupancy ratio in the protein and DNA channels. Moreover, the LBP algorithm is used to capture the histogram statistical information of protein channels based on the image coding strategy of the central pixel and peripheral pixels in the local mask, including 256-dimensional features (Xu et al., 2013). Finally, the 1096-dimension (836 + 4+256 = 1096) shallow features that combine the above features are fed into the BR classifier to construct HAR_Locator^S^ (Boutell et al., 2004). Above mentioned process can be shown in Figures 1B, C

#### 2.2.3 Multi-view abstract features derived from different layers of HARnet

Numerous papers have reported that abstract features derived from deep learning models are an effective supplement to shallow features for improving classification accuracy (Shao et al., 2017; Liu et al., 2019; Xue et al., 2020; Su et al., 2021; Ullah et al., 2021); most abstract features are from the last or penultimate layer of CNNs. Furthermore, abstract features from different depths of deep learning models also expressed infusive performance (Long et al., 2020). However, some problems pose challenges for complex, fine-grained, and large-sized IHC images (SeyedJafari and Hunger, 2017), such as information loss and feature degradation with deeper depth of CNNs, which demands a treatment for capturing more robust abstract features. In this work, a deep feature extraction network named HARnet based on hybrid attention modules and residual units was designed to capture multi-view abstract features in different layers for releasing the problems mentioned. The extraction of deep features is composed of two steps: in step 1, the HARnet is trained in an end-to-end training fashion and its architecture is shown at the bottom of Figure 1; in step 2, an IHC was described by the output of the four modules of HARnet, as part of which the basic image characteristics and abstract category properties were highlighted with an increasing depth of HARnet, i.e., three hybrid attention modules and the last Global Average Pooling (GAP) layer. The details about HARnet are described below.

#### 2.2.4 The HARnet based on hybrid attention modules and residual units

In natural image computer vision tasks, by stacking multiple convolutions, activation, and pooling layers, CNNs with multiple nonlinear functions can capture abstract image information after iterative parameter optimization (Ji et al., 2012; Chan et al., 2015; Mezgec and KoroušićSeljak, 2017). However, simple stacked convolutional networks are prone to sticking in gradient dispersion, network degradation, and poor performance due to complexity and fine-grained IHC images. Hence, the HARnet based on the **H**ybrid **A**ttention modules and **R**esidual unit was first developed to extract abstract features, and its properties can be summarized as follows: firstly, to effectively capture discriminant features of IHC images, the hybrid attention modules that fuse bottom-up top-down feedforward structure and multi-scale channel attention are introduced to focus on protein-target regions (Wang et al., 2017; Dai et al., 2021); secondly, the backbone network of HARnet was superimposed multiple residual units, and the gradient information can be preferably transmitted (He et al., 2016); finally, in the last layer of HARnet, the GAP layer rather than Fully Connection(FC) layer is employed, which not only releases the training burden and time but also avoids overfitting problems. The abstract features came from the three hybrid attention modules and the last GAP layer. The details of HARnet are displayed at the bottom of Figure 1.

The hybrid attention module is composed of three branches, among which the top two represent global information of IHC feature maps at multi-scale channel attention, the bottom one amplifies local region information through a fusion of bottom-up top-down feedforward structure, and the equation of hybrid attention module 2 (H2) is illustrated in Eqs 1–4. The key ideas of multi-scale channel attention are as follows: firstly, the local context $P\left(X\right)$ from pointwise convolution of the top pipeline was executed to express pixel-level discriminant features by varying spatial size; secondly, the global context $G\left(X\right)$ from global average pooling of every channel was described global view in whole feature maps; thirdly, the pixel-level local context $P\left(X\right)$ and the global view of the global context $G\left(X\right)$ enrich the feature information in the hybrid attention modules, as shown in Eqs 1, 2. Furthermore, local region features $B\left(X\right)$ in the bottom branch of H2 can be broken down into two steps: in step 1, residual units R are used to remove irrelevant information and further refine image abstract information, and the response of the receptive field is enhanced by performing maxpooling layer $M_{1}$ in a 3 × 3 local mask; step 2, after reaching the lowest resolution of feature maps, bilinear interpolation was executed to achieve the original resolution of feature maps. The sum of local context $P\left(X\right)$ and global context $G\left(X\right)$ is then normalized to (0, 1) through the Sigmoid function and the result is used as the weight to obtain the local discriminant features of $B\left(X\right)$ , as shown in Eqs 3, 4. The hybrid attention modules 1 and 3 (H1 and H3) have a structure that is similar to that of H2, with the primary exception being that H1 includes an additional bottom-up top-down feedforward layer in its bottom branch, and H3 has the identity map in its bottom branch. The process of refining and modifying of all attention modules are also displayed in Figure 1.

$$P\left(X\right)=p_{2}^{1}\left(p_{1}^{1}\left(X\right)\right)$$ (1)

$$G\left(X\right)=p_{2}^{2}\left(p_{1}^{2}\left(g\left(X\right)\right)\right)$$ (2)

$$B\left(X\right)=R_{2}\left(I_{1}\right.\left(M_{1}\left(R_{1}\left(X\right)\right)\right)$$ (3)

$$X^{\prime }=\sigma \left(P\left(X\right)+G\left(X\right)\right)*B\left(X\right)$$ (4)

Where X is the input, $X\in R^{C*H*W}$ , $X^{\prime }$ is the output of the hybrid attention module 2, $P\left(X\right)$ is the output of top branch of the hybrid attention module 2 by pointwise convolution, $G\left(X\right)$ is the output of second branch of the hybrid attention module 2, $B\left(X\right)$ is the output of bottom branch of the hybrid attention module 2, the filter size of $p_{1}^{1}$ and $p_{1}^{2}$ is $\left(\frac{c}{r},H,W\right)$ , r is 4, the filter size of $p_{2}^{1}$ and $p_{2}^{2}$ is $\left(C,1,1\right)$ , g is GAP layer, $M_{1}$ is MaxPooling, $I_{1}$ is interpolation function, σ is sigmoid function, $R_{1}$ and $R_{2}$ is Residual identity unit.

#### 2.2.5 Designing multi-classifier of HAR_Locator via SDA and BR

After the above processing, the integrated features were concatenated by combing abstract and shallow features. To avoid irrelevant information or redundant features, SDA is employed to select a more discriminative feature subset, and following that, the subset feature is fed into BR. In SDA, Wilks’λ statistical method was employed to judge iteratively discriminative features in the original feature space (Huang et al., 2003). The BR classifier uses seven One-vs-Rest (OvR) Support Vector Machine (SVM) classifiers, which are effective at determining class probability (Boutell et al., 2004). There are five subset classifiers in HAR_Locator: the ${HAR\_Locator}^{H_{i}\_S}$ ( i = 1, 2, 3) and HAR_Locator^G_S^ are trained by concatenating shallow and abstract features extracted from three hybrid attention modules, and the last GAP layer, the HAR_Locator^S^ is trained by shallow feature. After that, five classifiers were generated, that is, five sets of 1*7 confidence vectors from them were output to express a sample, just as in the section of Figures 1C, D.

#### 2.2.6 Multi-category multi-classifier decision system of HAR_Locator based on ANN

Taking up the above multi-classifier, an effective decision system is also helpful to further improve output results from the multi-classifier. In previous work, the output confidence was the mean of all classifier output probabilities, where the largest was the output label (Newberg and Murphy, 2008). However, this approach would weaken the representation of sample confidence. Based on the previous work, the ANN is designed with three hidden layers to get decision results; the hidden neurons of $n_{1}$ , $n_{2}$ , and $n_{3}$ are 256, 128, and 64 respectively. The ReLU activation function and Softmax function were adopted in this ANN (Cao et al., 2018). In the experiment, the output confidence of five predictors was concatenated into a 1*35-D vector to fit the network parameters. Finally, the test dataset was used to assess the performance of the HAR_Locator, as shown in the section of Figure 1D.

## 3 Results

In this section, the 10-fold cross-validating strategy is used to assess HAR_Locator and compare its performance to other predictors. The training process of HARnet is completed in 300 iterations using GPU parallel computing architecture and the Tensorflow-gpu2.4.0 deep learning framework. The BR classifiers were executed by Matlab software. Furthermore, the initial learning rate of the training process of HARnet is 0.001, which is multiplied by 0.1 around 60 epochs. Adam was utilized to optimize the parameters of HARnet.

### 3.1 HAR_Locator outperforms other baseline predictors

The baseline predictors were compared with HAR_Locator in performance evaluation indices like accuracy, precision, and recall (Newberg and Murphy, 2008; Xu et al., 2013; Jiao and Du, 2016), and the results showed that HAR_Locator ranks first among them. This was done to objectively and thoroughly verify the performance of HAR_Locator. The experimental results are shown in Table 2. From these three indexes, the predictor built by Newberg et al. through adopting Haralick features with different values of 8.0%, 7.63%, and 8.28% is inferior to the predictor built by Xu et al. through concatenating LBP features. However, the result of HAR_Locator was over 18.78%, 20.0%, and 19.11% higher than the method proposed by Xu et al. HAR_Locator significantly improves prediction performance, the main advances include the following. Firstly, the feature space composition of each image in HAR_Locator consists of two parts, namely, abstract features extracted from some modules of HARnet and shallow features based on the statistical method. After concatenating, the supervision ability of feature maps is significantly enhanced. Secondly, the HARnet built by hybrid attention modules and residual units was imported to extract abstract features, and the gradient information of IHC images can be effectively transmitted. The discriminative regions of feature maps in HARnet can be retained and enhanced, so abstract features with more supervisory and discriminative information have a better supplementary for shallow features and improve the performance of baseline predictors. Finally, a multi-category multi-classifier decision system is constructed to obtain the final output results of samples by fitting the most representative results in five basic predictors through ANN; it further improves the performance of HAR_Locator. It can be demonstrated that the HAR_locator has better experimental performance than baseline predictors based on shallow features by concatenating multi-view abstract features obtained from HARnet with shallow features derived from statistical methods.

**TABLE 2:** Comparison of HAR_Locator with baseline protein location predictors.

Model	Accuracy (%)	Precision (%)	Recall (%)
Newberg and Murphy (2008)	57.95	57.14	57.31
Xu et al. (2013)	65.95	64.77	65.59
HAR_Locator	**84.73**	**84.77**	**84.70**

### 3.2 HAR_Locator outshined other deep networks and derived models

**FIGURE 2:**
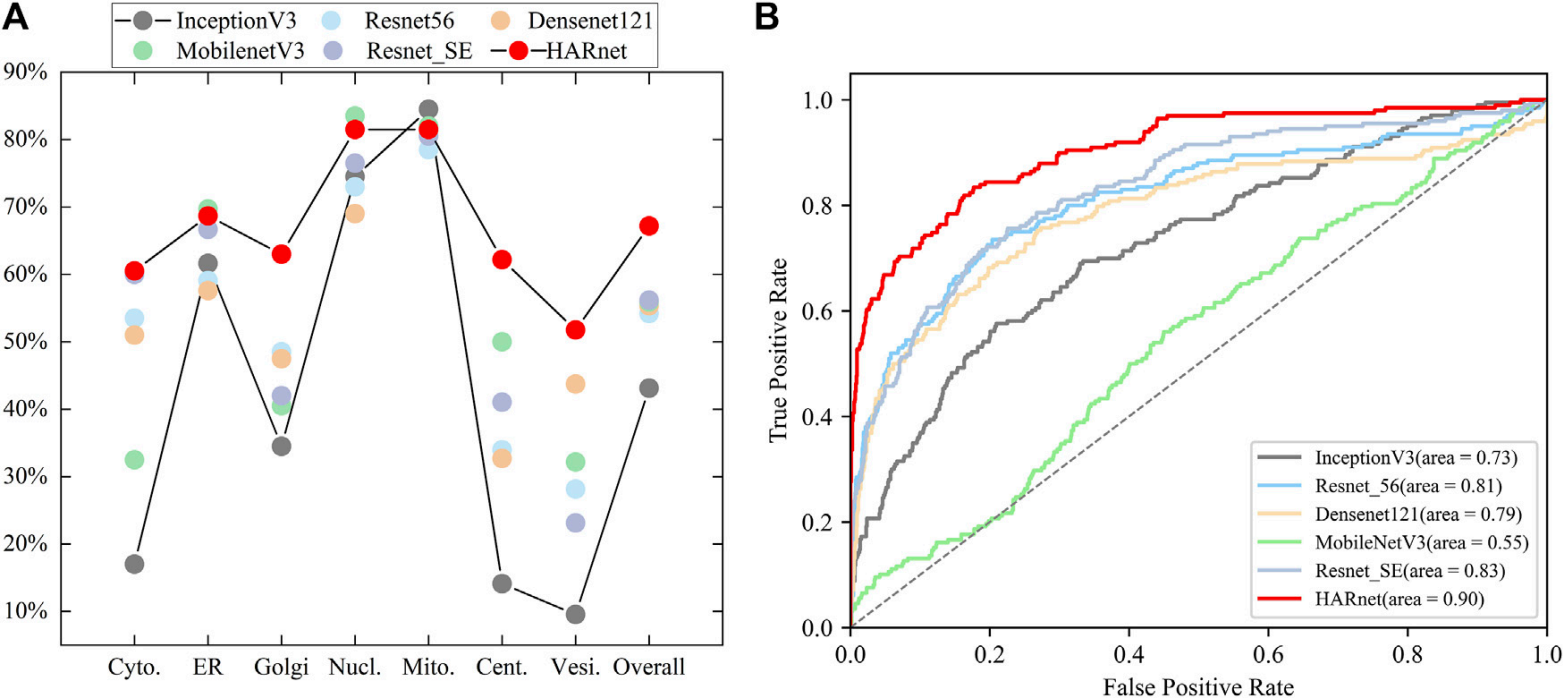
Visualization of performance evaluation in mainstream CNNs; **(A)** Single-class and overall predictive accuracy of protein subcellular location in different CNNs; **(B)** Receiver Operating Characteristic curve and Area Under Curve of various CNNs.

The performance of HARnet was confirmed when using 512*512 revolution IHC images as input and achieved the best performance in the mainstream network, such as InceptionV3, Resnet56, Densenet121, and MobilenetV3 (He et al., 2016; Szegedy et al., 2016; Huang et al., 2017; Hu et al., 2018; Howard et al., 2019). The experimental results of various CNNs were presented in Figure 2. The scatter plot of Figure 2A shows that HARnet outperformed other mainstream CNNs with 67.18% overall accuracy; multiple accuracies of different protein subcellular locations were higher than those of other CNNs, such as Golgi apparatus, Centrosome, Vesicles, and Cytosol. However, InceptionV3 achieved the last results in terms of accuracy. Among the protein subcellular locations involved, the prediction accuracy of the Nucleoli and Mitochondria exceeded that of the other subcellular locations in all models. Additionally, HARnet’s accuracy in the Nucleoli and Mitochondria was 81.5%, placing it second and third, respectively, among the CNNs mentioned. Correspondingly, the Receiver Operating Characteristic curve (ROC) was visualized to show fluctuations of various mainstream CNNs stimulated by different thresholds in Figure 2B. It can be seen that HARnet ranks first with an Area Under Curve (AUC) of 0.90. This shows that HARnet has the highest permutation ratio of positive samples to negative samples and the highest true validity of the test. In model structure, the advantages of HARnet can be summarized in the following aspects for IHC images: firstly, using a smaller filter size for the convolution kernel instead of stacking simply general convolution operation would conducive to capturing fine details and keeping feature information richness; secondly, the GAP layer rather than the FC layer was adapted to map three-dimensional deep features into a one-dimensional feature vector and sent into the Softmax layer, which is prone to overcome overfitting problems due to substantially increased training parameters. Expect for these, Resnet_SE refers to the network in which the attention module is replaced by the Squeeze Excitation (SE) module from the HARnet (Hu et al., 2018). The results of Resnet56, Resnet_SE, and HARnet show that the network with added attention modules has better performance than the models without attention modules, but the hybrid attention module utilized in HARnet allows more efficient acquisition for discriminative features of protein subcellular location patterns in IHC images.

In addition, some handcrafted predictors were constructed by feature fusion and BR classifier to recognize protein subcellular location. For example, the Resnet_SE^G^_BR in Table 3 is built from the following pipelines: firstly, similar to HARnet, the Resnet_SE is the backbone network built by residual units and embedded with SE modules; secondly, the G feature maps were derived from the GAP layer of trained Resnet_SE; finally, the Resnet_SE^G^_BR is successfully constructed by feeding the features into the SDA feature reduction dimension and BR classifier. The Resnet56^G^_BR is similar to the Resnet_SE^G^_BR except that the Resnet56^G^_BR consists of residual units only. Then, the Resnet56^G_S^_BR and the Resnet_SE^G_S^_BR are akin to the Resnet_SE^G^_BR; however, they do so by introducing shallow features. The derived models are inferior to the HAR_Locator with different experimental indices from Table 3. Some conclusions can be summarized as follows: firstly, it can be informed that the predictor trained by abstract features and shallow features can further improve its performance; secondly, the Resnet_SE achieved the best performance expected for HARnet in mentioned CNNs, and the derived models are similar to these experimental results; finally, the HARnet based on hybrid attention modules and residual units can present more effective feature maps, and the advanced HAR_Locator was proven.

**TABLE 3:** Comparison of results between the handcraft predictors models and sub-classifier of HAR_Locator.

Predictor	Accuracy (%)	Precision (%)	Recall (%)
Resnet56^G^_BR	59.68	59.27	59.36
Resnet56^G^ ^_S^_BR	74.56	73.22	74.38
Resnet_SE^G^_BR	57.84	57.39	57.18
Resnet_SE^G_S^_BR	72.67	71.56	72.47
HAR_Locator^A3_S^	82.90	81.61	82.81
HAR_Locator^G_S^	84.22	82.94	84.20
HAR_Locator	**84.48**	**84.43**	**84.42**

### 3.3 Comparison of abstract features and shallow features before and after concatenating

**FIGURE 3:**
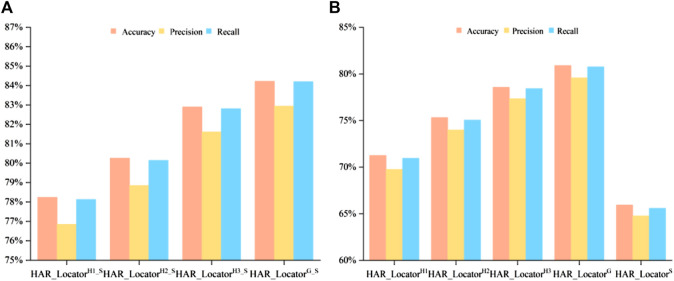
Comparison of multiple evaluation criteria after and before concatenating in multiple classifiers; **(A)** Classification performance after feature concatenation; **(B)** Experimental results before feature concatenation.

Some digital image characteristics of abstract features in HARnet, such as morphological local regions, edges, corners, and outlines, were collected to supplement the properties of shallow features. A crucial fact is that HAR_Locator performance may unquestionably be enhanced by concatenating shallow and deep features. In this part, multi-view features from HARnet were adopted to investigate the feature prediction effect under different module depths, with the results shown in Figure 3. It can be seen that the prediction performance obtained only by shallow features is inferior to that obtained by concatenating abstract and shallow features. For example, HAR_Locator^S^ can reach 65.95%, 64.77%, and 65.59% in accuracy, precision, and recall, while HAR_Locator^G_S^ constructed by connecting with GAP layer abstract features, can reach 84.22%, 82.94%, and 84.02%. The latter significantly improves the experimental results. The advanced performance mainly attributes to the following four aspects: firstly, the abstract features obtained from HARnet underwent multiple nonlinear function mappings and hybrid attention modules, which purposefully highlighted the subcellular location properties of the protein-target regions. Secondly, shallow features can be described by basic digital image characteristics, such as texture, inertia, isotropy, and spatial ratio, while abstract features express the structural components of the feature maps of the protein-target regions from different layers of HARnet and enrich the information richness of the protein subcellular location. Thirdly, as HARnet’s depth increases, feature maps transition from describing fundamental features of digital images to describing abstract features of the categories to which IHC images belong. The feature maps at different depths represent protein-target regions from different views and express the different generalization abilities of the abstract features at different layers of HARnet. Finally, the weakness caused by the poor supervisory and discriminative properties of shallow features can be addressed by combining shallow features with abstract features.

## 4 Discussion

### 4.1 Analyzing the composition of subset feature spaces after SDA

**FIGURE 4:**
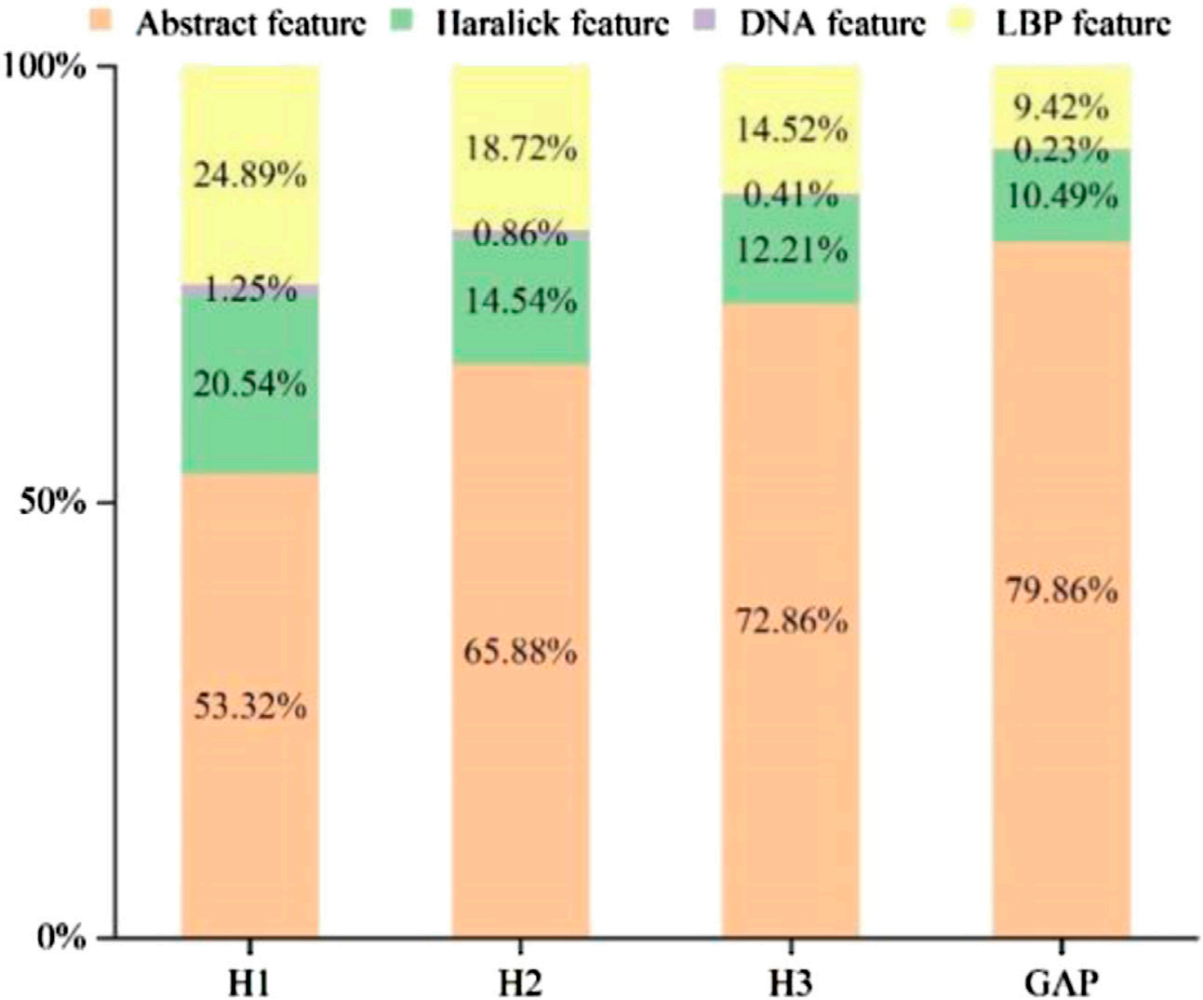
The feature ratios in different modules of HARnet.

A few ratios of various feature components were determined, as shown in Figure 4, to better understand the effect of the feature following SDA. Abstract features, Haralick features, LBP features, and DNA features all played a role in the integrated feature space. The figure respectively shows the feature selection distribution ratios of ${HAR\_Locator}^{H_{i}\_S}$ ( i = 1, 2, 3) and HAR_locator^G_S^ after SDA through 10-fold cross validation. For instance, the 3144-dimension feature of HAR_Locator^G_S^ was composed of 2048-dimension abstract features derived from the GAP layer of HARnet, 836-dimension Haralick features, 256-dimension LBP features, and 4-dimension DNA features. It reveals that shallow features make up 20.14% of the total, while abstract features account for 79.86%. In keeping with the aforementioned experimental results, we also discovered that the fraction of abstract characteristics increased as the depth of HARnet increased. Compared with the result of Table 2, the performance of some predictors constructed by shallow features, i.e., the automated framework proposed by Newberg et al. and the iLocator proposed by Xu et al., are far inferior to HAR_Locator. The complementarity between abstract features acquired by HARnet and shallow features can effectively enrich the information richness of protein subcellular location patterns and improve the model’s performance. Specifically, feature maps represent the various spatial information from the IHC image in various HARnet layers. The abstract representation and identifiability of feature maps are also significantly improved with increasing HARnet depth, which enhances the HAR_Locator’s generalization capabilities.

### 4.2 Investigating feature maps under different modules

**FIGURE 5:**
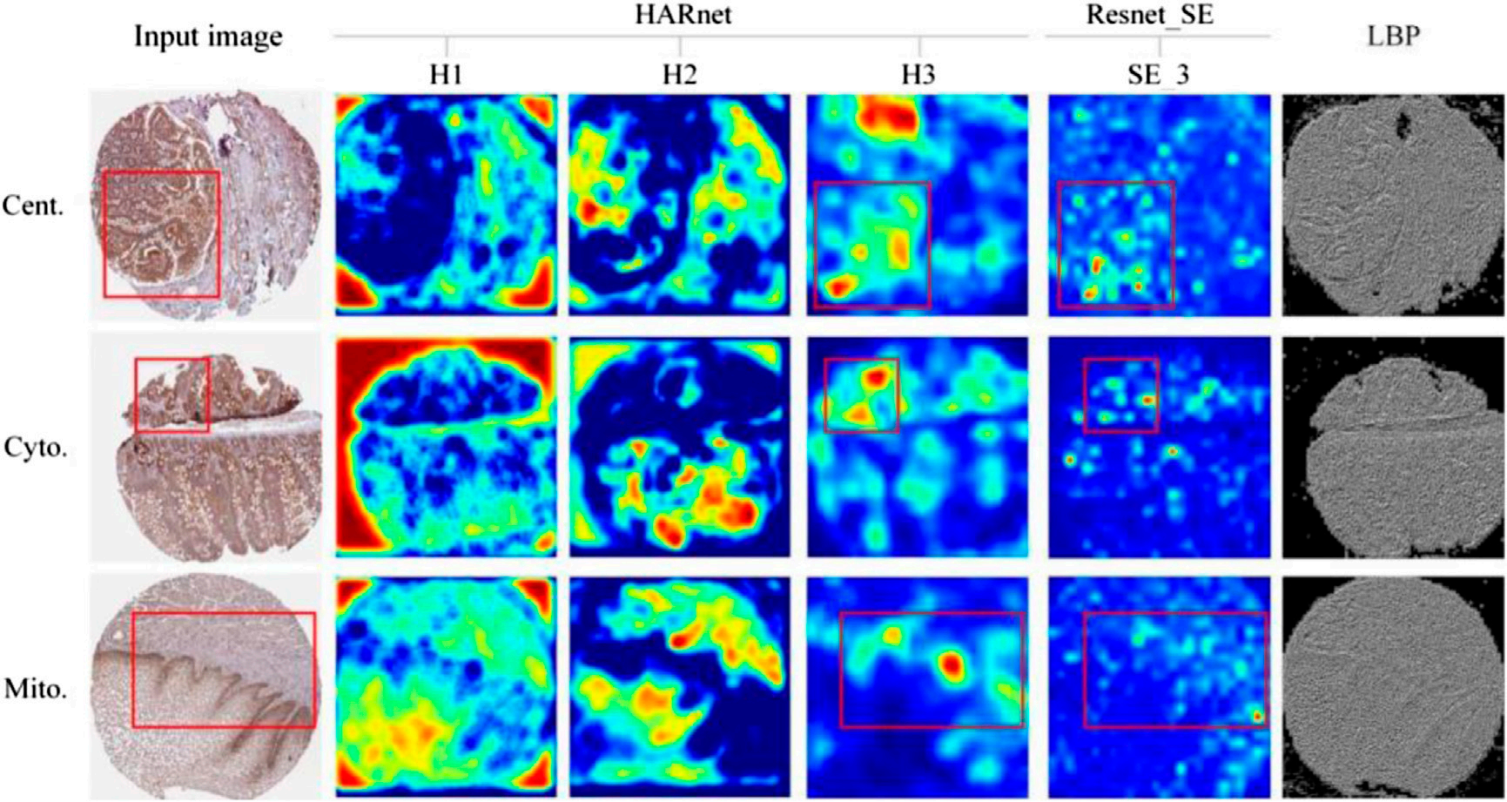
Feature maps visualization in different modules.

The outputs of a few attention modules and LBP were represented in Figure 5 to help more easily comprehend the specifics of feature maps under various modules. H1, H2, and H3 are the outputs of three hybrid attention modules of HARnet, SE_3 is the output of Resnet_SE in the third module of the Resnet_SE, and the last column is texture image under LBP. As can be seen from Figure 5, each row of the visualization represents the subcellular location pattern of one protein IHC image under different modules, namely, Centrosome, Cytosol, and Mitochondria. The second to fourth columns show that the protein-target regions in the feature map are gradually highlighted with the deepening of HARnet. Specifically, the H1 feature maps tend to express the contours and edges of protein-target regions; the H2 feature maps gradually started to focus on protein-target regions, but it was not inaccurate; and the H3 feature maps correctly identified the protein-target regions and displayed the target abstract morphology and protein highlight properties. As can be seen in the red box of Figure 5, the feature maps of SE_3 in Resnet_SE can highlight some protein-target regions, but they are weaker than H3 from HARnet. Meanwhile, the protein-target regions exhibited by the texture features in LBP are ambiguous. These investigations show that the high-level abstract features derived from HARnet are a potential addition to shallow features and can capture abstract information for boosting model performance. Also noteworthy is the fact that H3 typically captures local abstract information of the IHC image, whereas H2 typically captures global concrete information of the image. This further demonstrates how the integrated feature space collected from the HARnet’s multi-view layers may be mutually complementary and enhance the HAR_Locator’s robustness and generalizability.

## 5 Conclusion

In this study, an exact and effective model called HAR_Locator has been developed for predicting protein subcellular location. Concerning the complex and fine-grained IHC images, an integrated feature space made up of multi-view abstract features from HARnet and shallow features derived from statistical methods was used to improve information richness, supervision, and discriminant, which is helpful to increase performance. The HARnet assembled by hybrid attention modules and residual units was designed to spotlight discriminative regions of protein-target subcellular location patterns in IHC images, and aim to capture more robust abstract features from different layers; moreover, multiple sub-classifiers constructed by different depth abstract features and shallow features were adopted to output the probability that IHC images belong to the subcellular location; finally, a decision system based on ANN has been embraced to produce a nondestructive decision result. The experimental results reveal that HAR_Locator can achieve 84.73% prediction accuracy in the benchmark dataset from HPA, which is better than other baseline models’ performance. HAR_Locator participated in multi-view feature maps of HARnet and significantly improved feature richness and discriminant, in contrast to other baseline models based on shallow features and a last or penultimate abstract feature. The effectiveness of the combination of the hybrid attention modules and residual units has been verified by quantitative and qualitative analysis. Taken together, it shows that HAR_Locator is effective for accurately analyzing protein subcellular location patterns. Naturally, there is also a crucial problem that necessitates consideration. The remaining feature dimensions of abstract features after SDA were decreased substantially compared with the original abstract feature dimension, which indicates that the original feature space has great redundancy. The subsequent research strategy for protein subcellular pattern analysis will therefore involve screening more discriminant feature maps from various layers.

## Data Availability

The original contributions presented in the study are included in the article/Supplementary Material, further inquiries can be directed to the corresponding author.
